# Prediction of the Growth Rate of Early-Stage Lung Adenocarcinoma by Radiomics

**DOI:** 10.3389/fonc.2021.658138

**Published:** 2021-04-15

**Authors:** Mingyu Tan, Weiling Ma, Yingli Sun, Pan Gao, Xuemei Huang, Jinjuan Lu, Wufei Chen, Yue Wu, Liang Jin, Lin Tang, Kaiming Kuang, Ming Li

**Affiliations:** ^1^ Department of Radiology, Huadong Hospital Affiliated With Fudan University, Shanghai, China; ^2^ Department of Thoracic Surgery, Huadong Hospital Affiliated With Fudan University, Shanghai, China; ^3^ Department of Radiology, The Second Affiliated Hospital of Chongqing Medical University, Chongqing, China; ^4^ Dianei Technology, Shanghai, China

**Keywords:** pulmonary nodules, tomography, X-ray computer, radiomics, volume doubling time, machine learning

## Abstract

**Objectives:**

To investigate the value of imaging in predicting the growth rate of early lung adenocarcinoma.

**Methods:**

From January 2012 to June 2018, 402 patients with pathology-confirmed lung adenocarcinoma who had two or more thin-layer CT follow-up images were retrospectively analyzed, involving 407 nodules. Two complete preoperative CT images and complete clinical data were evaluated. Training and validation sets were randomly assigned according to an 8:2 ratio. All cases were divided into fast-growing and slow-growing groups. Researchers extracted 1218 radiomics features from each volumetric region of interest (VOI). Then, radiomics features were selected by repeatability analysis and Analysis of Variance (ANOVA); Based on the Univariate and multivariate analyses, the significant radiographic features is selected in training set. A decision tree algorithm was conducted to establish the radiographic model, radiomics model and the combined radiographic-radiomics model. Model performance was assessed by the area under the curve (AUC) obtained by receiver operating characteristic (ROC) analysis.

**Results:**

Sixty-two radiomics features and one radiographic features were selected for predicting the growth rate of pulmonary nodules. The combined radiographic-radiomics model (AUC 0.78) performed better than the radiographic model (0.727) and the radiomics model (0.710) in the validation set.

**Conclusions:**

The model has good clinical application value and development prospects to predict the growth rate of early lung adenocarcinoma through the combined radiographic-radiomics model.

## Introduction

Lung cancer has the highest incidence rate of all cancers in China and worldwide. The incidence rate and mortality rate of lung cancer in 2018 were 11.6% and 18.4%, respectively (1). In recent years, the incidence rate of lung adenocarcinoma was the highest among lung cancers, accounting for 60% of primary lung cancers, and lung adenocarcinoma is the most common histological type of lung cancer (2, 3). With the widespread use of low-dose CT screening, the increasing early-stage lung cancer are discovered (4). A pulmonary nodule is defined as a rounded opacity that is well or poorly defined measuring up to 3 cm in diameter (5). Although the current guidelines issued by the National Comprehensive Cancer Network (NCCN) (6, 7), Fleischner Society (8), American College of Chest Physicians (ACCP) (9), and the Asian Consensus (10) differ in some respects regarding the diagnosis and treatment of and follow-up strategies for pulmonary nodules, most guidelines are based on radiographic features, such as the type and size of pulmonary nodules. Lung adenocarcinoma is an inert tumor compared with squamous cell carcinoma and small cell carcinoma (11), although it is not uncommon for lung nodules to rapidly grow from the early stage to advanced stage in a short period. However, such nodules are usually followed up with CT examinations, and the best treatment time may be missed. Following the diagnosis and treatment methods recommended by previous guidelines for such fast-growing malignant nodules may lead to untimely diagnosis and treatment of patients, resulting in serious disease. Among all early-stage lung cancers (T1N0M0 stage), the choice of treatment and the prognosis of patients are sometimes quite different in clinical practice. Among similarly sized pulmonary nodules, the prognosis of aggressive lung adenocarcinoma is significantly worse than that of inert nodules.

Radiomics extracts engineering features related to morphology, histogram, intensity, and texture and integrates knowledge from multiple fields of imaging (image interpretation), computers (quantitative feature extraction) and machine learning (model establishment and evaluation) (12, 13). Radiomics can reveal a large amount of invisible, high-dimensional information with potential clinical value hidden behind the image. Currently, radiomics has achieved good results in the diagnosis of benign and malignant pulmonary nodules (14, 15). However, there are few radiomics methods for the prediction of the growth rate of pulmonary nodules. Therefore, the aim of this study was to establish a radiomics-based model for the prediction of the growth rate of early lung adenocarcinoma to assist in clinical decision-making.

## Materials and Methods

### Data Collection

#### Methods

From January 2012 to June 2018, patients with lung adenocarcinoma confirmed by pathology who had two or more thin-layer CT images were selected. The inclusion criteria were as follows: 1) nodule diameter<3 cm; 2) more than 2 preoperative thin-layer CT scans with an interval of more than 30 days (15); 3) pulmonary nodules confirmed by pathology as lung adenocarcinoma; and 4) all cases with stage T1N0M0. The exclusion criteria were as follows: 1) preoperative surgery or chemoradiotherapy treatment; 2) no complete clinical data; and 3) unclear image due to respiratory motion and other factors and nodules with details that could not be displayed.

In total, 407 pulmonary nodules from 402 patients (mean age 58.45, 22-84 years) were included in this study. The cases were randomly divided into the training set and validation set at a ratio of 8:2. Only no-contrast CT images were included in this study. If the patients had more than two CT images, the first CT scan image and the last preoperative CT scan image were selected. The average interval between the two CT images was 567.56 days (range 30-2813), with a median of 397 days. This study was approved by the institutional review committee of our hospital, and patient informed consent was not required.

### Radiographic Features and Postoperative Pathological Evaluation

The first CT images of all patients were independently evaluated by two chest radiologists with 6 and 12 years of chest CT reading experience (evaluation conditions: window width, 1500 Hounsfield units [HU]; window position, -700 HU). Any discrepancies in the interpretation between the observers were resolved by a consensus. The CT findings of each lesion were analyzed, including (1) the lesion location, (2) lesion type (pure ground glass, partial solid nodule, or solid nodule), (3) lesion size, (4) margin (clear or blurred), (5) nodule shape (round, oval, or irregular), (6) pleural attachment, including pleural tag and indentation (absent or present), (7) bubble (absent or present), (8) bronchiole change (absent or present), (9) vascular change (absent or present), (10) and lobulation (absent or present). Solid nodule as a nodule that completely obscures the entire lung parenchyma within it. Part-solid GGN are those having sections that are solid in this sense, and pure GGN are those with no solid parts (16). Vascular changes were defined as the thickening and twisting of blood vessels through the lesion or aggregation of vessels surrounding the lesion (17). Bronchiole changes were defined as enlargement, distortion, or obstruction of the bronchus through the lesion. In the analysis of interobserver reliability, the type (pure GGN, part GGN or solid nodules) were compared between 2 observers, diagnostic concordance was assessed by unweighted kappa values. Two senior pathologists (chest pathologists with more than 5 and 10 years of working experience) evaluated and reviewed the lung tissue according to the classification of lung adenocarcinoma by the International Lung Cancer Research Association, American Thoracic Society and European Respiratory Society (2).

### Evaluation of Growth Rate

The tumor volume doubling time (VDT) is a key parameter used to distinguish fast-growing tumors from slow-growing tumors (18). Since few studies investigated the growth rate of pulmonary nodules, we defined the growth rate of pulmonary nodules according to previous studies. Most studies defined pulmonary nodules with a VDT >400 days and VDT ≤400 days as slow-growing and fast-growing pulmonary nodules, respectively (11, 19).

### Image Acquisition, Nodule Segmentation and VDT Acquisition

All CT scans were performed with one of the four scanners (GE Discovery CT750HD, 64-slice LightSpeed, VCT, Somatom Definition Flash; Somatom Sensation 16). The detailed scan and reconstruction parameters are listed in **Table 1**.

**Table 1:** CT scanning parameters.

	GE Discovery CT750 HD	Light Speed VCT	Somatom Definition Flash	Somatom Sensation 16
Tube voltage	120 kVp	120 kVp	120 kVp	120 kVp
Tube current	200 mA	200 mA	200 mA	200 mA
Pitch	0.984:1	0.984:1	1.0	0.8
Collimation	0.625 mm*64	0.625 mm*64	0.6 mm*64	0.75 mm*16
Rotation time	0.5 s/rot	0.5 s/rot	0.33 s/rot	0.35 s/rot
SFOV	50 cm	50 cm	50 cm	50 cm
Slice thickness of reconstruction	1.25 mm	1.25 mm	1 mm	1/1.5 mm
Slice interval of reconstruction	1.25 mm	1.25 mm	1 mm	1/1.5 mm
Reconstruction algorithm	STND	STND	Medium sharp	Medium sharp
Number of nodules	103	138	67	99

An open-source medical image processing and navigation software 3D slicer (version 4.8.0, Brigham and Women’s Hospital) was used to manually delineate the volume of interest (VOI) of the 814 nodules by a radiologist with 6 years of experience with chest CT interpretation; then, the VOI was confirmed by another radiologist with 12 years of chest CT interpretation who corrected the boundary of each nodule to avoid the influence of vessels, bronchus, pleura and other structures outside the nodule to the greatest extent possible (20). Finally, all CT data of the VOI of all nodules were exported in NII (desensitization format) for the following analysis.

The volume doubling time (VDT) refers to the time required to calculate the volume doubling based on an exponential growth model (21). Two CT scan images were selected for each patient to analyze and calculate the VDT as follows: the first CT image was selected as the scan image from the first CT scan in our hospital, and the second image was the last preoperative CT scan image. The following formula was used to calculate the VDT:

$\text{VDT}=(\text{volume}\;\text{doubling}\;\text{time})=\frac{(T1-T0*log2}{log\;(V1/V0)},$where *V*0 and *V*1 represent the volumes at *T*1 (time 1- the second examination date) and *T*0 (time 0 – the baseline examination date).

### Feature Extraction and Repeatability Analysis of Radiomics

#### Feature Extraction

Pyradiomics Toolkit (Version 2.1.0, https://github.com/Radiomics/pyradiomics) was used to extracted the radiomics features, including the first-order features based on the CT value or the pixel value of the preprocessed image, the shape descriptor features used to describe the shape and size, the gray-level cooccurrence matrix (GLCM), the gray-level run lengths matrix (GLRLM), the gray level size zone matrix (GLSZM), and the gray level dependence matrix (GLDM), to describe the internal and surface texture of the lesion. In total, 1218 radiomics features were extracted from each lesion (22).

#### Repeatability Analysis

**Figure 1:**
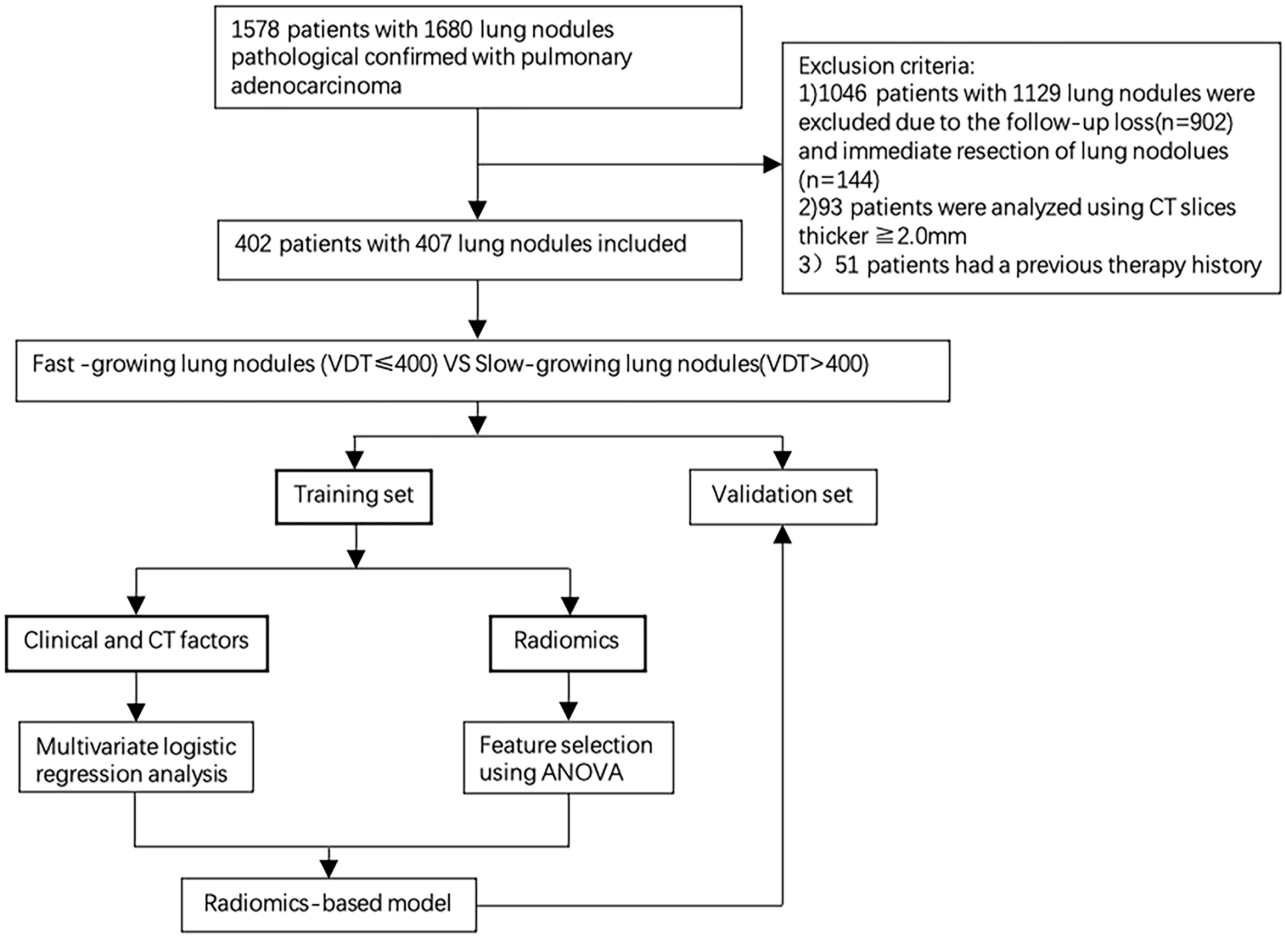
The workflow of the study.

The specific method was as follows: 60 nodules were randomly selected for independent segmentation by a radiologist with 6 years of experience with chest CT interpretation. One month later, the radiologist repeated the same procedure. Then, the interclass correlation coefficient (ICC) was assessed to analyze the correlation of the 1218 features extracted from these 60 nodules. Finally, features with an ICC >0.80 were selected and included in the follow-up study (23) (**Figure 1**).

### Feature Selection, Model Establishment, and Verification

#### Feature Selection

Data collected in practice often have many missing values, duplicate values, and abnormal values, but the final value of the model depends on the amount of useful information. Therefore, the Python 3.7.1 software is used to normalize the feature with Min-Max scaling. Finally, to remove the irrelevant features to prevent over fitting and enhance the robustness of the model, an ANOVA was used to perform a univariate analysis of each radiomics feature, and the radiomics feature that had the most significant impact was selected.

#### Establishment and Verification of the Model

First, in the training set, the statistically significant differences in the radiographic features between the two groups were analyzed by a univariate analysis, and a multivariate analysis was performed to confirm the independent radiographic predictors that can predict the growth rate of pulmonary nodules. A fast, distributed and high-performance gradient promotion framework (Light Gradient Boosting Machine, LightGBM) based on a decision tree algorithm was used to build a radiomics-based model. Finally, the AUC value was used to evaluate the effectiveness of the model.

### Statistical Analysis

All statistical analyses were performed using R statistical software (version 3.4.3; http://www.Rproject.org) and a commercially available software program (SPSS 23.0 for Windows; SPSS, Chicago, IL, USA), and the qualitative data are described as n (%). A chi square test or Fisher’s exact test was used to analyze the categorical variables, and an independent-samples t-test or Kruskal Wallis test was used to analyze the continuous variables. The ROC curve, area under curve (AUC) and precision recall (P-R) curve were used to evaluate the predictive effectiveness of the model, and *P*<0.05 indicated statistical significance. The integrated discrimination improvement (IDI) was used to measure the difference in model performance (24), and an IDI>0 indicates that the performance of the model is improved.

## Results

### Comparison of Radiographic Features Between Slow- and Fast-Growing Nodules

In total, 407 pulmonary nodules from 402 patients with two or more CT images were collected from January 2012 to June 2018, including 41 AIS 42, 158 MIA and 207 IPA. According to the VDTs of the nodules, the 407 nodules were divided into the following two groups: fast-growing nodules (n=77, 18.9%; average VDT=221.78 days) and slow-growing nodules (n=330, 81.1%; average VDT=1722.21 days). In total, 325 cases (fast-growing nodules: n=61; slow-growing nodules: n=264) and 82 cases (fast-growing nodules: n=16; slow-growing nodules: n=66) were randomly divided into the training sets and validation set at a ratio of 8:2. There was no statistically significant difference in the distribution of the clinical and radiographic features between the training sets and validation set, except for the nodule shape and bronchiole change. However, after multivariate analysis in the training set, the two factors were not included in the subsequent analysis. The radiographic features evaluated by the two chest radiologists were almost perfect agreement (unweighted kappa-values, 0.898). The complete patient profiles in the two sets are shown in **Table 2**.

**Table 2:** Patient information for the training and validation sets.

Demographic and Clinical Characteristic	Training set (n=325)	Validation set (n=82)	*p*
Sex			0.385
Male	193 (59.4%)	53 (64.6%)	
Female	132 (40.6%)	29 (35.4%)	
Age (years)	57.575±11.652	57.939±11.048	0.532
Size (cm)	0.892±0.498	0.890±0.505	0.928
Location			0.882
Right upper lobe	90 (27.7%)	25 (30.5%)	
Right middle lobe	51 (15.7%)	15 (18.3%)	
Right lower lobe	93 (28.6%)	21 (25.6%)	
Left lower lobe	28 (8.6%)	5 (6.1%)	
Left lower lobe	63 (19.4%)	16 (19.5%)	
Smoking history			0.531
Never smoker	22 (6.8%)	4 (4.9%)	
Current or former smoker	302 (93.2%)	78 (95.1%)	
Family history of cancer			1.000
Present	5 (1.5%)	1 (1.2%)	
Absent	320 (98.5%)	81 (98.8%)	
Margin			0.116
Clear	64 (19.7%)	10 (12.2%)	
Blurred	261 (80.3%)	72 (87.8%)	
Type			0.849
Pure ground glass	130 (40.0%)	30 (36.6%)	
Partial solid nodule	130 (40.0%)	35 (42.7%)	
Solid nodule	65 (20.0%)	17 (20.7%)	
Shape			0.025*
Round	137 (42.2%)	30 (36.6%)	
Oval	105 (32.3%)	39 (47.6%)	
Irregular	83 (25.5%)	13 (15.9%)	
Pleural attachment			0.446
Present	72 (22.2%)	15 (18.3%)	
Absent	253 (77.8%)	67 (81.7%)	
Bubble			0.827
Present	33 (10.2%)	9 (11.0%)	
Absent	292 (89.8%)	73 (89.0%)	
Bronchiole change			0.014*
Present	37 (11.4%)	2 (2.4%)	
Absent	288 (88.6%)	80 (97.6%)	
Vascular change			0.164
Present	37 (11.4%)	14 (17.1%)	
Absent	288 (88.6%)	68 (82.9%)	
Lobulation			0.419
Present	55 (16.9%)	17 (20.7%)	
Absent	270 (83.1%)	65 (79.3%)	
Growth rate			0.878
Fast-growing nodules	61 (18.8%)	16 (19.5%)	
Slow-growing nodules	264 (81.2%)	66 (80.5%)	

Age and size are shown as the mean ± standard deviation; other data are shown as the number of patients, with the percentage in parentheses. The P value is derived from the univariate association analyses between clinical parameters and the growth rate of pulmonary nodules.

*p value < 0.05.

### Feature Selection and Establishment of the Radiomics Model

In total, 575 robust radiomics features (ICC: 0.8002-0.9777) were selected for the follow-up analysis. In total, 62 radiomics features (the first 10 radiomics features are shown in **Table 3**) were selected. The details of 62 radiomics features were included in the **Supplement Data**.

**Table 3:** Top 10 imaging features after feature screening.

Class	Feature name
First-order features	wavelet-HHH_firstorder_Variance
Gray-level cooccurrence matrix	wavelet-LHH_glcm_ClusterProminence wavelet-HHH_glcm_Contrast wavelet-HHH_glcm_ClusterTendency’ wavelet-HHH_glcm_ClusterProminence wavelet-HHH_glcm_DifferenceVariance wavelet-HHH_glcm_SumSquares
Gray-level run lengths matrix	wavelet-HHH_glrlm_GrayLevelVariance
Gray level dependence matrix	wavelet-HHH_gldm_SmallDependenceHighGrayLevelEmphasis wavelet-HHH_gldm_GrayLevelVariance

In the univariate analysis, four CT factors were statistically significantly associated with the growth rate of pulmonary nodules (**Table 4**).Then, According to the results of the multivariate analysis, the nodule type (pure GGN, part GGN, or solid nodules) was ultimately identified as an independent risk factor for the prediction of the growth rate of nodules (**Table 5**) and was included in the model establishment.

**Table 4:** Comparison of fast-growing and slow-growing cases in the training set.

Demographic and Clinical Characteristic	Fast-growing nodules (n=61)	Slow-growing nodules(n=264)	P
Sex			0.351
Male	33 (54.1%)	160 (60.6%)	
Female	28 (45.9%)	104 (39.4%)	
Age (years)	56.885±11.986	58.966±11.562	0.238
Size (cm)	0.997±0.529	0.868±0.489	0.030*
Location			0.297
Right upper lobe	14 (23.0%)	76 (28.8%)	
Right middle lobe	7 (11.5%)	44 (16.7%)	
Right lower lobe	18 (29.5%)	75 (28.4%)	
Left lower lobe	9 (14.8%)	19 (7.2%)	
Left lower lobe	13 (21.3%)	50 (18.9%)	
Smoking history			0.180
Never smoker	7 (11.5%)	15 (5.7%)	
Current or former smoker	54 (88.5%)	249 (94.3%)	
Family history of cancer			0.943
Present	1 (1.6%)	4 (1.5%)	
Absent	60 (98.4%)	260 (98.5)	
Margin			0.154
Clear	16 (26.2%)	48 (18.2%)	
Blurred	45 (73.8%)	216 (81.8%)	
Type			0.001*
Pure ground glass	17 (27.9%)	113 (42.8%)	
Partial solid nodule	21 (34.4%)	109 (41.3%)	
Solid nodule	23 (37.7%)	42 (15.9%)	
Shape			0.036*
Round	17 (27.9%)	120 (45.5%)	
Oval	23 (37.7%)	82 (31.1%)	
Irregular	21 (34.4%)	62 (23.5%)	
Pleural retraction			0.605
Present	12 (19.7%)	60 (22.7%)	
Absent	49 (80.3%)	204 (77.3%)	
Bubble lucency			0.396
Present	8 (13.1%)	25 (9.5%)	
Absent	53 (86.9%)	239 (90.5%)	
Bronchiole change			0.384
Present	5 (8.2%)	32 (12.1%)	
Absent	56 (91.8%)	232 (87.9%)	
Vascular change			0.358
Present	5 (8.2%)	32 (12.1%)	
Absent	56 (91.8%)	232 (87.9%)	
Lobulation			0.011*
Present	17 (27.9%)	38 (14.4%)	
Absent	44 (72.1%)	226 (85.6%)	

Age and size are shown as the mean ± standard deviation; other data are shown as the number of patients, with the percentage in parentheses. The P value is derived from the univariate association analyses between clinical parameters and the growth rate of pulmonary nodules.

*p value < 0.05.

**Table 5:** Multivariate analysis of the radiographic features.

Characteristic	OR (95% CI)	*p*
Size	0.992 (0.932-1.056)	0.806
Type	1.701 (1.112-2.603)	0.014
Shape	1.319 (0.903-1.926)	0.152
Lobulation	1.405 (0.653-3.021)	0.384

OR, odds ratio; CI, confidence interval.

### Evaluation of the Predictive Effectiveness of the Radiomics Models

**Figure 2:**
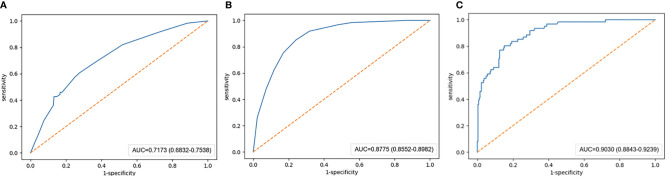
AUC value of the training set. **(A)** The AUC value of the radiographic model with the training set is 0.717. **(B)** The AUC value of the radiomics model with the training set is 0.876. **(C)** The AUC value of the combined radiographic-radiomics model with the training set is 0.903.

**Figure 3:**
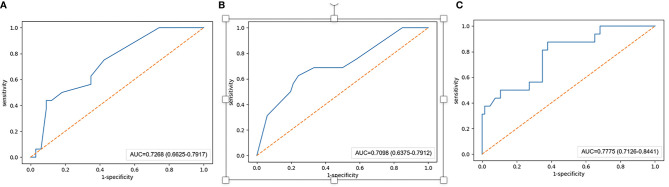
AUC value of the validation set. **(A–C)** The AUC value of the radiographic model with the validation set is 0.727. **(B)** The AUC value of the radiomics model with the validation set is 0.710. **(C)** The AUC value of the combined radiographic-radiomics model with the validation set is 0.778.

In the training set, the AUCs of the radiographic model, the radiomics model and the combined radiographic-radiomics model were 0.717 (95%CI: 0.683-0.754), 0.876 (95%CI: 0.855-0.898) and 0.903 (95%CI: 0.884-0.924), respectively (**Figure 2**). And the recall of the radiographic model, the radiomics model and the combined radiographic-radiomics model were 60.7% (precision 71.7%), 85.3% (precision 81.5%) and 80.3% (precision 83.7%), respectively. In the validation set, the AUCs of the radiographic model, the radiomics model and the combined radiographic-radiomics model were 0.727 (95%CI: 0.663-0.792), 0.710 (95%CI: 0.638-0.754) and 0.778 (95%CI: 0.713-0.844), respectively (**Figure 3**). And the recall of the radiographic model, the radiomics model and the combined radiographic-radiomics model were 43.8% (precision 80.5%), 62.5% (precision 74.4%) and 87.5% (precision 65.6%). Compared with the radiographic model, the IDI value of the combined radiographic-radiomics model was 0.086(versus 0), and the difference was statistically significant (z statistic, P<0.05). Compared with the radiomics model, the IDI value of the combined model was 0.094 (versus 0), indicating a statistically significant difference (z statistic, P<0.05). Finally, the results showed that the growth rate of early lung adenocarcinoma could be predicted more effectively by the combined model.

## Discussion

In this study, 407 lung nodules were followed up by chest CT examinations over a long period, and the value of the radiomics features and radiographic features in predicting the growth rate of early-stage lung adenocarcinoma was analyzed. Finally, we established a combined radiographic-radiomics model that can better predict the growth rate of lung adenocarcinoma, with an AUC of 0.778 (95%CI: 0.713-0.844) and a recall of 87.5% (precision 65.9%).

Previously, the prediction of the growth of pulmonary nodules mainly depended on radiographic features (25). However, these CT features are subjective and inaccurate. In this study, we not only analyzed the radiographic features but also combined radiomics features to include more information and establish a more convenient and feasible prediction model, and the model finally achieved good performance.

In this study, the univariate analysis of the clinical data and CT features showed that there were statistically significant differences in the size, type, shape and lobulation of nodules between the fast-growing and slow-growing nodules (*P*<0.05). However, after the multivariate analysis, only the type (pure GGN, part GGN or solid nodules) significantly differed between the two groups. In total, 77 fast-growing pulmonary nodules were identified, including 19 cases (24.7%) of pure GGN, 28 cases (36.3%) of partial GGN, and 30 cases (39.0%) of solid nodules. From this result, we can get a general conclusion that aggressive nodules may be more commonly observed in patients with solid nodules and have a lower VDT; these results are consistent with previous studies (26, 27). Additionally, Oda S et al. found that the growth rate of solid nodules is usually faster than that of part GGNs and pure GGNs (26, 28). We speculate that this finding may be due to most solid components of malignant nodules being mainly proliferative stacks of tumor cells. In this study, although the size, shape and lobulation of the nodules in the univariate analysis were statistically significantly different, these features can predict the growth rate of pulmonary nodules remains to be determined. Kobayashi et al. (29) reported that the nodule size may be robustly associated with the growth of pulmonary nodules; however, this study did find the correlation after multivariate analysis, the reasons may include the following aspects: first, this study only used a univariate analysis and should not be considered authentic. Second, this study used manual measurement of the nodule size to judge the growth of pulmonary nodules; however, the presence of subjective factors, such as inaccurate measurement and error, require a more rigorous analysis.

Radiomics is widely used in clinical research because it can excavate a large amount of invisible information with great clinical value and has achieved good results (20, 30). Due to the instability of the features, a repeatability analysis was carried out. Most studies are based on the assumption that the features are redundant, thus reducing the number of radiomics features used in modeling research. However, each feature may affect the training accuracy to a certain extent. Therefore, to avoid overfitting the model and enhance the robustness, this study adopted data normalization and an ANOVA, and 62 useful features were finally selected for model establishment. According to the theory of radiomics, the features we extracted can reflect the spatial heterogeneity, microenvironment and gene expression of tumors (31). LightGBM is a newly developed gradient lifting algorithm framework based on a decision tree algorithm that can further optimize the tree model and is more conducive to clinical application (32). Therefore, we established a combined radiographic-radiomics model that can better distinguish the growth rate of pulmonary nodules and proved the feasibility of the modeling method. Yooh et al. (33) evaluated 52 lung adenocarcioma patients and demonstrated the potential of margin-related radiomics feature to predict tumor doubling times in lung adenocarcinoma, however, we finally decided that dividing the lung nodules into slow- and fast-growing nodules according to the VDT, because it can be more conducive to the management of nodules in clinical, and has the potential to develop a personalized follow-up strategy for patients (19).

There are certainly some underlying limitations to this study. First, this study was a single-center retrospective study that lacked external validation data, and prospective studies are needed to assess the robustness and practical clinical value of the combined model. Second, due to the different reconstruction cores, the CT acquisition protocol is not standardized, which may have had a potential impact on the extraction of the radiomics features. However, all images included in the current study were thin-slice CT image to minimize these variabilities (34). Third, the model achieved good results with the training set but lower results in the validation set, which may be caused by the limited amounts of samples in the study. In the future, we aim to improve the efficacy and feasibility of this model through multicenter data, standardized CT scanning parameters and a larger sample.

In summary, this study analyzed the value of CT features and radiomics features in the diagnosis of lung adenocarcinoma. Considering its practicability and accuracy, we established a combined model that can better predict the growth rate of pulmonary nodules and assist in clinical decision-making.

## Data Availability Statement

The raw data supporting the conclusions of this article will be made available by the authors, without undue reservation.

## Ethics Statement

The studies involving human participants were reviewed and approved by the Ethic Committee of the Huadong Hospital Affiliated with Fudan University. Written informed consent for participation was not required for this study in accordance with the national legislation and the institutional requirements.

## Author Contributions

ML and YS conceived the presented idea. LJ collected the data. LJ, YW, PG, LT, KK, and XH analyzed the data. MT and WM drafted the manuscript. All authors reviewed the manuscript, and ML made corrections to the manuscript. All authors contributed to the article and approved the submitted version.

## Funding

This study was supported by the National Nature Science Foundation of China 61976238 (ML), “Future Star” of famous doctors’ training plan of Fudan University, Science and Technology Planning Project of Shanghai Science and Technology Commission 20Y11902900 (ML). Medical Imaging Key Program of Wise Information Technology of 120, Health Commission of Shanghai 2018ZHYL0103 (ML).

## Conflict of Interest

KK was employed by Dianei Technology, Shanghai.

The remaining authors declare that the research was conducted in the absence of any commercial or financial relationships that could be construed as a potential conflict of interest.
