# Supplementary material for: Prediction of the Growth Rate of Early-Stage Lung Adenocarcinoma by Radiomics
Source: Front Oncol. 2021 Apr 15;11:658138. doi: 10.3389/fonc.2021.658138 (PMC8082461; doi:10.3389/fonc.2021.658138)
Supplement: Supplementary file 1 [file Table_1.docx]

**Supplementary data**

Table 1.The final 62 radiomics features

| Class | Feature name |
| --- | --- |
| First-order features | log-sigma-1-0-mm-3D_firstorder_90Percentile  wavelet-LLL_firstorder_Variance  original_firstorder_Range  log-sigma-3-0-mm-3D_firstorder_Variance  original_firstorder_Variance  original_firstorder_Maximum  wavelet-HLL_firstorder_Maximum  wavelet-LLL_firstorder_RobustMeanAbsoluteDeviation  log-sigma-3-0-mm-3D_firstorder_RobustMeanAbsoluteDeviation  wavelet-LLL_firstorder_MeanAbsoluteDeviation  original_firstorder_MeanAbsoluteDeviation  wavelet-LLL_firstorder_InterquartileRange  log-sigma-3-0-mm-3D_firstorder_InterquartileRange  log-sigma-4-0-mm-3D_firstorder_10Percentile  log-sigma-4-0-mm-3D_firstorder_Minimum  original_firstorder_RobustMeanAbsoluteDeviation  log-sigma-3-0-mm-3D_firstorder_MeanAbsoluteDeviation  wavelet-LLL_firstorder_Range  wavelet-HHH_firstorder_Variance |
| Gray-level cooccurrence matrix | wavelet-LHH_glcm_ClusterProminence  wavelet-HHH_glcm_Contrast  wavelet-HHH_glcm_ClusterTendency  wavelet-HHH_glcm_ClusterProminence  original_glcm_ClusterTendency  wavelet-LLL_glcm_ClusterTendency  original_glcm_ClusterProminence  log-sigma-1-0-mm-3D_glcm_Autocorrelation  log-sigma-3-0-mm-3D_glcm_ClusterProminence  log-sigma-3-0-mm-3D_glcm_ClusterTendency  log-sigma-3-0-mm-3D_glcm_SumSquares  wavelet-LLL_glcm_SumSquares  original_glcm_SumSquares  wavelet-LLL_glcm_Autocorrelation  wavelet-HHH_glcm_DifferenceVariance  wavelet-HHH_glcm_SumSquares |
| Gray-level run lengths matrix | wavelet-HHH_glrlm_GrayLevelVariance  log-sigma-1-0-mm-3D_glrlm_HighGrayLevelRunEmphasis  log-sigma-1-0-mm-3D_glrlm_ShortRunHighGrayLevelEmphasis  wavelet-LLL_glrlm_GrayLevelVariance  log-sigma-3-0-mm-3D_glrlm_GrayLevelVariance  log-sigma-1-0-mm-3D_glrlm_LongRunHighGrayLevelEmphasis  original_glrlm_GrayLevelVariance  wavelet-LLL_glrlm_LongRunHighGrayLevelEmphasis  original_glrlm_LongRunHighGrayLevelEmphasis  log-sigma-3-0-mm-3D_glrlm_ShortRunHighGrayLevelEmphasis  log-sigma-3-0-mm-3D_glrlm_HighGrayLevelRunEmphasis |
|  |  |
| Gray level dependence matrix  Gray level size zone matrix | wavelet-HHH_gldm_SmallDependenceHighGrayLevelEmphasis  wavelet-LHH_gldm_SmallDependenceHighGrayLevelEmphasis  log-sigma-1-0-mm-3D_gldm_HighGrayLevelEmphasis  wavelet-LLL_gldm_GrayLevelVariance  log-sigma-3-0-mm-3D_gldm_GrayLevelVariance  original_gldm_GrayLevelVariance  wavelet-LLL_gldm_HighGrayLevelEmphasis  log-sigma-3-0-mm-3D_gldm_HighGrayLevelEmphasis  wavelet-HHH_gldm_GrayLevelVariance  log-sigma-1-0-mm-3D_glszm_SmallAreaHighGrayLevelEmphasis  wavelet-LLL_glszm_GrayLevelVariance  log-sigma-5-0-mm-3D_glszm_SizeZoneNonUniformity  wavelet-HHH_glszm_GrayLevelVariance  log-sigma-3-0-mm-3D_glszm_GrayLevelVariance  original_glszm_GrayLevelVariance  log-sigma-1-0-mm-3D_glszm_HighGrayLevelZoneEmphasis |
